# Novel* SUFU* Frameshift Variant Leading to Meningioma in Three Generations in a Family with Gorlin Syndrome

**DOI:** 10.1155/2019/9650184

**Published:** 2019-07-28

**Authors:** Gustav Askaner, Ulrikke Lei, Birgitte Bertelsen, Alessandro Venzo, Karin Wadt

**Affiliations:** ^1^Department of Plastic Surgery, Hospital South West Jutland, Esbjerg, Denmark; ^2^Department of Dermatology and Allergy, Gentofte Hospital and Rigshospitalet, Copenhagen University Hospital, Copenhagen, Denmark; ^3^Center for Genomic Medicine, Rigshospitalet, Copenhagen University Hospital, Copenhagen, Denmark; ^4^Department of Plastic Surgery and Burns Treatment, Rigshospitalet, Copenhagen University Hospital, Copenhagen, Denmark; ^5^Department of Clinical Genetics, Rigshospitalet, Copenhagen University Hospital, Copenhagen, Denmark

## Abstract

Gorlin syndrome is mainly caused by pathogenic germline variants in the tumour suppressor genes* PTCH1* and* SUFU*, both regulatory genes in the hedgehog pathway. However, the phenotypes of patients with* PTCH1* and* SUFU* pathogenic variants seem to differ. We present a family with a frameshift variant in the* SUFU* gene c.954del, p.Asn319Thrfs*∗*42 leading to meningiomas and multiple basal cell-carcinomas.

## 1. Introduction

Gorlin syndrome (GS) or nevoid basal cell carcinoma syndrome is a rare autosomal dominant genetic disorder. A recent English study [1] found a prevalence of GS in 1:30.827, which potentially is an underestimation as individuals with a mild clinical phenotype might not be recognised. The syndrome was first described by Gorlin and Goltz in 1960 as consisting of multiple nevoid basal cell-carcinomas (BCCs) and odontogenic keratocysts of the jaw and bifid ribs [2]. Other phenotypical findings have since been linked to GS and currently more than 100 manifestations have been linked to the disorder [3]. As a consequence of the highly variable clinical phenotype, the clinical diagnosis is made from five major criteria and eight minor criteria [3]. Around 85-90% of affected individuals carry pathogenic variants in either* PTCH1* or* SUFU* and the phenotypic variability is closely linked to the gene affected [4].* PTCH1* and* SUFU* are tumour suppressor genes in the hedgehog pathway. In accordance with Knudson's two-hit hypothesis, a germline pathogenic variant of a tumour suppressor gene is a first hit. After the first hit it only takes a somatic mutation in the other allele (the second hit) to completely disable the tumour suppressor protein, leading to a drastically increased probability of developing a tumour from this cell line.

The majority of patients with GS have loss of function (LOF) pathogenic variants in the* PTCH1* gene. A smaller fraction of patients with GS have pathogenic variants in the* SUFU* gene, which predominantly have been LOF variants [4]. The* PTCH2* gene, a close homolog of* PTCH1*, has also been associated with GS [5]. As some large studies did not find any significant difference between individuals with germline* PTCH1* or* SUFU* variants, regarding age of onset of BCC or total number of BCCs, it has been debated whether there is a genotype-phenotype correlation regarding BCCs in GS [4, 6, 7]. However, these studies only included few individuals with pathogenic variants in the* SUFU* gene. As germline variants only have a first hit effect, skin phenotype and environmental factors such as exposure to UV-radiation play an important role in the risk of BCC development. BCC is a common skin tumour in the Caucasian population, hence making it difficult to identify a statistically significant difference. There is however consensus that the genotype influences the phenotype for other tumorous disorders and malformations in GS [6, 8].* PTCH1* pathogenic variants are associated with jaw keratocyst and a higher rate of skeletal anomalies compared to pathogenic variants in the* SUFU* gene, where to-date keratocysts of the jaw have not been reported [8].* SUFU* pathogenic variants are associated with a higher rate of meningiomas and medulloblastomas compared to* PTCH1* [4, 9]. Studies have also shown that some of the variability seen in GS might be due to multilayered genetic variants in the hedgehog pathway related genes [10].

## 2. Case Presentation

**Figure 1:**
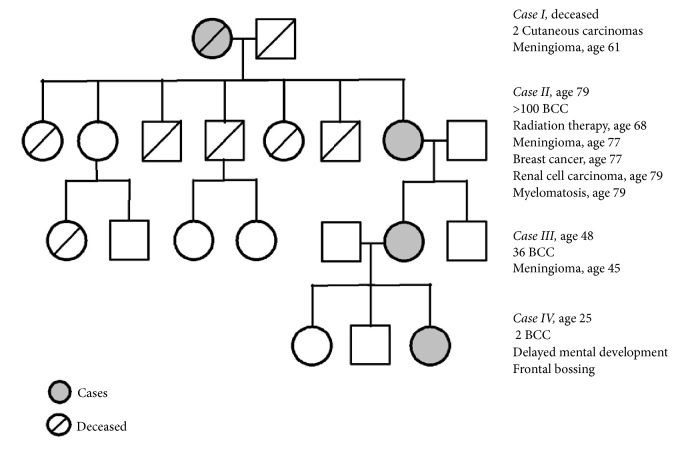
Pedigree. Cases are marked with grey and their most relevant medical history is listed to the right.

We present a family with four cases of four directly descending generations (Figure 1). All four cases, three of whom had a history on meningioma, carry a 1-bp deletion (c.954del) in* SUFU* resulting in a frameshift and a premature stop codon (p.Asn319Thrfs*∗*42).

### 2.1. Case I

**Figure 2:**
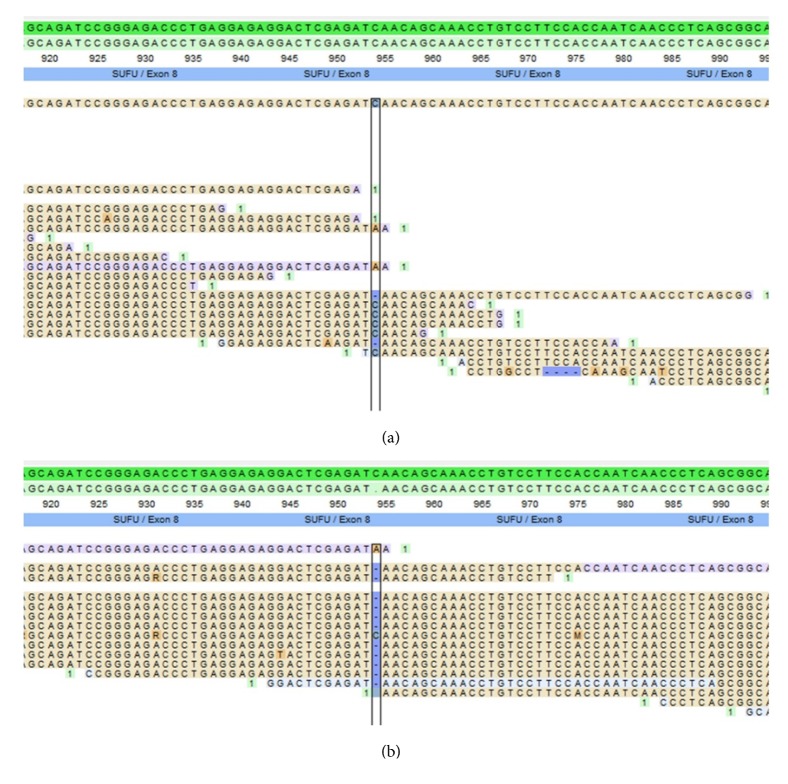
Reads of the meningioma tissue of case I and III. (a) Case I, showing the* SUFU* c.954delC variant but no LOH. (b) Case III, showing the* SUFU* c.954delC variant and LOH.

Deceased female: the patient had a meningioma removed at the age of 61, which was pathologically verified. Tumour tissue from the meningioma was genetically tested using panel-based NGS. The frameshift mutation was found in* SUFU* but it did not show loss of heterozygosity (LOH) (Figure 2). Because of low quality of DNA from the formalin-fixed and paraffin-embedded tissue and thus low coverage of sequencing reads, a somatic LOF mutation on the other allele could not be excluded. The patient had 2 confirmed cutaneous carcinomas at the age of 71 and 73. Additional medical records are sparse but mentions diabetes mellitus.

### 2.2. Case II

**Figure 3:**
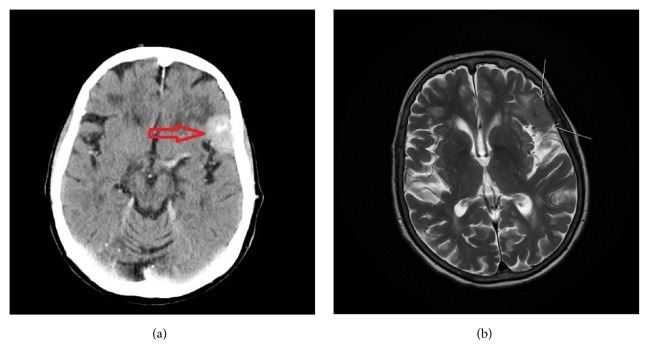
Imaging of meningioma of case II. (a) Contrast CT in the axial plane visualizing the meningioma (red arrow). (b) T2 weighted MRI in the axial plane visualizing the same meningioma (white arrows).

79-year-old female: at the age of 68 years the patient received 40,5 Gy in radiation therapy for a BCC located to the left lateral canthus. The patient developed a meningioma, located at the lateral part of the left sphenoidal bone, at the age of 77 years (Figure 3). Pathology description of the meningioma showed a WHO grade I meningioma. She has had multiple BCCs, >100 from the age of 52 years. At the age of 68 years the patient was suspected of GS and an X-ray of the skull revealed calcification of falx cerebri but no jaw cysts. The patient had a history of breast cancer (invasive ductal carcinoma) at age 77 years and just recently, at the age of 79 years, the patient was diagnosed with myelomatosis and clear cell renal cell carcinoma. The patient was otherwise known with hypertension and noninsulin dependent diabetes mellitus.

### 2.3. Case III

48-year-old female: the patient developed meningioma located at the left temporal region at the age of 45 years. Pathology showed an atypical meningioma WHO grade II. Genetic testing using panel-based NGS of tissue from the meningioma showed LOH in the* SUFU* gene (Figure 2). The patient has had multiple BCCs, a total of 36 from the age of 34 years and to current age. The patient had a gallstone operation at the age of 24 years and was otherwise healthy.

### 2.4. Case IV

25-year-old female: the patient has had two BCCs, the first at the age of 22 years. Genetic testing using panel-based NGS of germline DNA identified a 1-bp deletion (c.954del) in* SUFU.* The patient had frontal bossing and mild delayed mental development. A recent MRI of the cerebrum showed no meningiomas and the patient was otherwise healthy.

## 3. Discussion

All our study subjects had the germline frameshift variant in* SUFU* c.954del, p.Asn319Thrfs*∗*42 and no pathogenic variants of* PTCH1*. Cases I, II, and III had a history of meningioma and cases II, III, and IV had confirmed early onset/multiple BCCs. Cases II, III, and IV each fulfilled two major criteria for GS (Figure 1) but did not fulfill any of the minor criteria. Case II had calcification of falx cerebri and multiple BCCs. Case III had multiple BCC and a first-degree relative with GS. IV had early onset of BCCs and a first-degree relative with GS. However, none of the patients in our study had undergone any further examination to identify skeletal abnormalities or other occult minor criteria. Some researchers question whether* SUFU* variant leads to GS or if it leads to a similar syndrome, with some of the same phenotypes but more centred around dermatological manifestations and childhood medulloblastoma [8]. In accordance with Evans* et al*. [3] we identify cases II, III, and IV as having GS because they have two major criteria and a pathogenic variant in* SUFU*. The phenotypes of our study subjects were consistent with larger observational studies of patients with GS stratified according to genotype [4, 7].

Though none of our study subjects had a history of medulloblastoma, a germline pathogenic variant in* SUFU* has previously been strongly associated with medulloblastomas [9, 11]. Medulloblastoma associated with pathogenic* SUFU* variants usually emerges earlier than nonhedgehog related medulloblastomas [9]. A study of 131 cases of childhood medulloblastomas [12] found germline pathogenic variants in* SUFU* in 8 cases (6%). Evans* et al*. [4] showed medulloblastomas in three out of nine patients with GS and* SUFU* germline pathogenic variants.

Choudry* et al*. [13] showed that seven out of seven patients with GS associated medulloblastomas treated with postoperative radiation therapy developed a secondary intracranial tumour, and meningiomas are the most common secondary brain tumours after cranial radiation therapy [14]. Case II in our study had a history of radiotherapy to treat a BCC in the same area as the meningioma developed and it is therefore possible that the meningioma in case II was radiation induced. As postoperative radiotherapy is also commonly used in the treatment of medulloblastomas if GS is not suspected [15], it can be difficult to differentiate the primary meningiomas from the radiation induced meningiomas in patients with pathogenic variants of* SUFU*. Meningiomas are also the most common primary, nonglial intracranial tumours [16].

The LOH of* SUFU* in the meningioma in our study (case III) shows that it is highly likely that the development of meningioma in this subject was due to the inherited* SUFU* pathogenic variant, consistent with the two-hit hypothesis. As there was no LOH in the meningioma of case I, it is uncertain if the development of this meningioma was caused by the pathogenic* SUFU* variant, but a LOF mutation on the other allele could not be excluded.

Aavikko* et al*. [17] presented a similar family to ours, with a missense* SUFU* variant, c.367C>T, p.Arg123Cys, leading to a significant affection of the function of the SUFU protein. Their study presented five family members with meningiomas and LOH of the* SUFU* gene was detected in all the meningiomas. The study subjects had no BCCs or other manifestations of GS. The authors hypothesized that the increased risk of meningioma is related to missense variations and due to some remaining activity of SUFU the cases did not develop medulloblastomas. In addition, Huq* et al*. [8] presented a case with a* SUFU* c.1365+2T>A variant resulting in a truncated protein. The study subjects, four siblings, had a history of multiple BCCs, first BCC 40-55 years old. They all had calcification of falx cerebri. One of the subjects had a history of meningioma, but no genetic testing of the meningioma was mentioned. Kijima* et al*. [18] have also presented a case where a nonsense* SUFU* c.550C>T, Gln184Ter variant resulted in amongst other conditions: medulloblastoma, multiple BCCs, and meningioma. However, they did not find LOH of the* SUFU* gene from the meningioma and the patient had received radiation therapy for the medulloblastoma. It could be argued, in line with the hypothesis of Aavikko* et al*. [17], that the severity of the* SUFU* variant dictates the phenotype; i.e., a truncated protein without function leads to a condition with multiple BCCs and increased risk of developing meningiomas and medulloblastomas, whereas partial function is associated with an increased risk of developing meningiomas. If this holds true, our study family should have had a high risk of developing childhood medulloblastomas. However, another possibility is that the difference in phenotype between these case series was due to multilayered variants in genes related to the hedgehog pathway as hypothesized by Onodera* et al*. [10], radiation exposure, and possibly pigmentation genes. In Onodera et al.'s study of four patients with GS and* PTCH1* variants they found that all patients had additional variants in genes related to the hedgehog pathway [10].

## 4. Conclusion

Our study presents four cases of GS in one family due to a frameshift variant in* SUFU*, three of which have developed meningiomas but no medulloblastomas. Of the two genetically tested meningiomas, one had LOH of the* SUFU* gene. The third meningioma was not genetically tested, and this patient had previously been exposed to radiation therapy. The correlation between pathogenic* SUFU* variants and meningiomas has been described by other [8, 17, 18]. Comparing our cases with these similar cases, it is not evident that it is solely the type of* SUFU* variant that dictates the phenotype in these patients. Further studies are needed to be able to prove or dismiss this correlation. However, it is intricate as meningiomas are common, especially in patients with therapeutic radiation exposure and the development of BCCs is also largely influenced by other factors, e.g., skin type and exposure to ionizing radiation. Another possibility of explaining the difference in phenotype in these patients could be due to multilayered variants in genes related to the hedgehog pathway.
